# Effects of Chemical and Biological Fungicide Applications on Sexual Sporulation of *Rhizoctonia solani* AG-3 TB on Tobacco

**DOI:** 10.3390/life14030404

**Published:** 2024-03-18

**Authors:** Yingmei Yang, Jie Zhang, Jiduo Yan, Lianjin Zhao, Li Luo, Chengyun Li, Genhua Yang

**Affiliations:** State Key Laboratory for Protection and Utilization of Bio-Resources in Yunnan, Yunnan Agricultural University, Kunming 650201, China; 15587109656@163.com (Y.Y.); zxy_1902@126.com (J.Z.); zc_0510@126.com (J.Y.); zhao12345620220714@163.com (L.Z.); luoli550612@163.com (L.L.)

**Keywords:** tobacco target spot disease, *Thanatephorus cucumeris*, survival time, stimulated sporulation, pathogenicity, circadian rhythm, infection regulation, biological control

## Abstract

*Rhizoctonia solani* AG-3 TB primarily causes tobacco target spot disease by producing a large number of sexual spores. However, inducing sexual spore formation under in vitro conditions has been challenging, impeding further research on its control. In this study, field experiments were conducted to assess the effects of different concentrations of chemical and biological fungicides on the production of sexual spores of *R. solani* AG-3 TB on tobacco plants. The results demonstrated that four chemical fungicides (propiconazole-morpholine guanidine, bordeaux mixture, thiophanate-methyl, and mancozeb) significantly induced sexual spore formation. Among them, increasing the concentrations of the first three fungicides resulted in an increase in the number of sexual spores, while increasing the concentration of mancozeb led to a decrease in spore count. The pathogenic fungus produced more sexual spores during the night than during the day. Temperature, humidity, and light conditions influenced spore production. Additionally, the infection rate of sexual spores was directly proportional to their concentration and inoculation time, but their survival time did not exceed 6 h in vitro. Importantly, *Streptomyces rectiolaceus* A8 significantly suppressed sexual spore formation, achieving an 83.63% control efficacy in the field and producing antimicrobial substances against *R. solani* AG-3 TB. In conclusion, appropriate concentrations of chemical fungicides can induce sexual spore formation, while A8 can inhibit their production, showing potential value for controlling tobacco target spot disease.

## 1. Introduction

**Figure 1:**
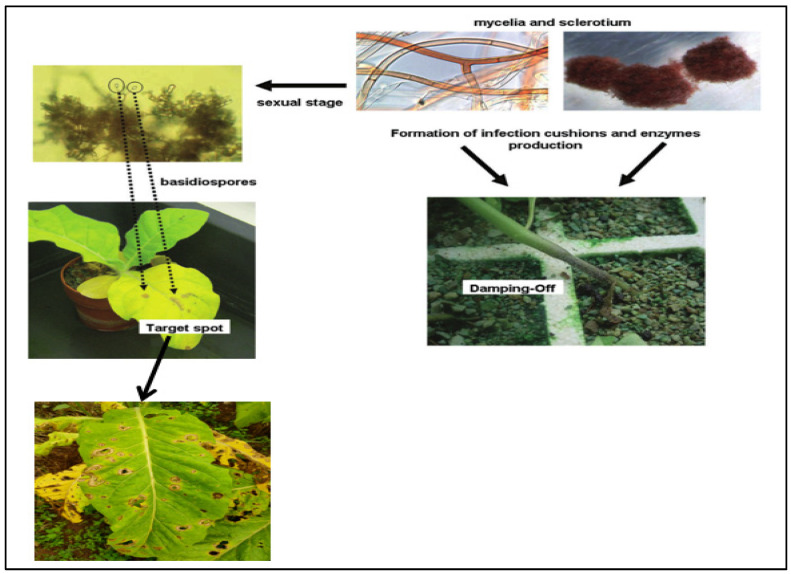
Disease cycle of *Rhizoctonia solani* and *Thanatephorus cucumeris* on tobacco.

*Rhizoctonia solani* AG-3 TB, causing tobacco target spot disease, has historically induced significant yield and quality losses worldwide [1,2,3]. Figure 1 depicts the life cycle and disease progression of a tobacco pathogen [4]. The mycelium of the pathogen grows in the soil and forms sclerotia [5]. Sclerotia are robust structures composed of tightly interwoven hyphae, are capable of surviving under harsh environmental conditions for up to three years, and under favorable conditions can germinate and produce new mycelium [6,7,8]. The pathogen can also produce basidiospores through sexual reproduction, which can be windborne, infect new tobacco plants, and form infection cushions near the roots of the tobacco plants [9]. The pathogen uses enzymes produced at the infection site to breach the cell walls of the plant, thus invading the interior of the tobacco plant [10]. The seedling stage of tobacco is particularly susceptible to target spot disease. Once infected, the seedling’s roots rot, the stems become thin, and it may develop into damping-off disease, leading to seedling death [4].

*R. solani* AG-3 TB exhibits significantly different infection patterns on different host plants. For example, when infecting tobacco, it produces large numbers of sexual spores and rapidly spreads through the tobacco plants, causing significant losses [11]. However, when infecting other crops such as potato, tomato, and rice, it does not produce sexual spores but instead generates abundant hyphae, spreading through the crops [12]. This phenomenon is highly uncommon in *Rhizoctonia* disease. The sexual spores of *R. solani* AG-3 TB are particularly rare, which does not favor further study of the sexual spores of *R. solani* AG-3 TB. The pathogen is the multinucleated *Rhizoctonia*, which does not produce asexual spores; its sexual spores are still difficult to induce under ordinary conditions. For example, the viability of sexual spores is particularly fragile in vitro, so they are difficult to induce indoors [13]. Researchers around the world have explored a range of methods for inducing sexual spore production in *R. solani*, including soil induction, medium induction, plant tissue induction, and hyphal anastomosis [14,15,16,17]. Currently, there are no reports in China on the use of chemical fungicides to induce the sexual spore production of *R. solani*. However, the study by Kangatharalingam and Carson provided evidence that chemical fungicides can induce fungal sexual spore production [18]. In our preliminary field investigation of tobacco target spot disease, we observed that despite the prevalent use of chemical fungicides by tobacco farmers aiming to control the disease, such practices do not always yield the expected outcomes. In fact, it was noted that the overuse of these fungicides could potentially contribute to the exacerbation of the disease conditions. Additionally, tobacco target spot disease is primarily caused by the sexual spores of *R. solani* AG-3 TB.

Environment parameters, particularly temperature, light, air, and humidity, are relevant factors for fungal spore production and survival [19,20,21,22]. Temperatures can directly affect the production of sexual or asexual spores by the fungus, or they can indirectly affect the diffusion and distribution of spores in the host plant. Under natural conditions, the optimal light conditions for fungal spore formation are consistent with the light conditions required by the host plant, and there is a significant correlation between light intensity and spore production [23]. Shew et al. reported that tobacco leaves produced basidiospores when RH = 100%, temperature at 20–26 °C [24].

Biological control is considered an environmentally friendly method for plant disease control [25,26]. For example, *Streptomyces* spp. have shown outstanding effectiveness in biological control against various fungal pathogens [27]. *Streptomycetes* can suppress or kill pathogens by producing bioactive molecules such as enzymes that disrupt fungal cell walls and compounds with fungicidal activity [28,29]. Foliar sprays of bioactive compounds produced by *Streptomyces* spp. can control symptoms caused by pathogens, although *Streptomyces* spp. inhabiting plant leaves is rare [30]. The ability of *Streptomyces* spp. to stimulate systemic plant resistance makes them promising candidates for the effective biological control of plant diseases. In our laboratory, an antagonistic actinomycete strain A8 isolated from soil was identified as *Streptomyces rectiolaceus* based on morphological features and phylogenetic analysis. The fermentation filtrate (FL) of A8 has a significant inhibitory effect on microorganisms, including Bacillus, Acetobacter, Agrococcus, and *R. solani*. At the same time, the actinomycete cell suspension (AC) of A8 has a significant control effect on rice sheath blight. Therefore, in this study, A8 was selected to control tobacco-targeted spot disease caused by the sexual spores of *R. solani* AG-3 TB.

## 2. Materials and Methods

### 2.1. Chemical Fungicides Induce Sexual Sporulation in R. solani AG-3 TB

The experiment was conducted during a complete growing season, covering the period from June to September 2023. It was conducted in fields where tobacco-targeted spot disease had occurred in previous years. The trial followed a fully randomized design with seven treatments and three replications. Each plot measured 10 m × 1.4 m (50 holes), and there were a total of 21 plots. Two-month-old tobacco seedlings (Yunyan 87) were transplanted into the field. To induce sexual sporulation, the tobacco leaves were sprayed with six chemical fungicides (18% propiconazole-morpholine guanidine, 70% thiophanate-methyl, and 70% mancozeb were provided by Guizhou Daoyuan Biotechnology Co., Ltd. in Anshun City, China. Bordeaux solution, 50% carbendazim and 50% dimethomorph were obtained from Tongzhou Zhengda Pesticide Chemical Co., Ltd. in Nantong City, China) at various concentrations after natural disease occurrence.

The treatments included the following: treatment p: spraying propiconazole-morpholine guanidine at the recommended concentration (1.25 g/L), twice the concentration (2.50 g/L), and four times the concentration (5.00 g/L); treatment b: spraying bordeaux solution at the recommended concentration (1.67 g/L), twice the concentration (3.34 g/L), and four times the concentration (6.68 g/L); treatment t: spraying thiophanate-methyl at the recommended concentration (1.00 g/L), twice the concentration (2.00 g/L), and four times the concentration (4.00 g/L); treatment m: spraying mancozeb at the recommended concentration (1.00 g/L), twice the concentration (2.00 g/L), and four times the concentration (4.00 g/L); treatment c: spraying carbendazim at the recommended concentration (1.00 g/L), twice the concentration (2.00 g/L), and four times the concentration (4.00 g/L); treatment d: spraying dimethomorph at the recommended concentration (0.63 g/L), twice the concentration (1.26 g/L), and four times the concentration (2.52 g/L); treatment CK: control group sprayed with an equal amount of water.

The dosages of the above agents were applied according to the manufacturer’s recommended usage. After seven days, the same dosage of pesticide was applied again to maintain its effectiveness. After one month, six randomly selected diseased tobacco leaves were collected from each treatment group. A 1 cm × 1 cm area of each tobacco leaf was stained with safranine and KOH dye, and the morphology was observed under a microscope. The number of basidiospores, sporogenic structures, and fruiting bodies were recorded. This observation process was repeated every 30 min until the end of the observation period.

### 2.2. Circadian Rhythm and Conditions of Sexual Sporulation

**Figure 2:**
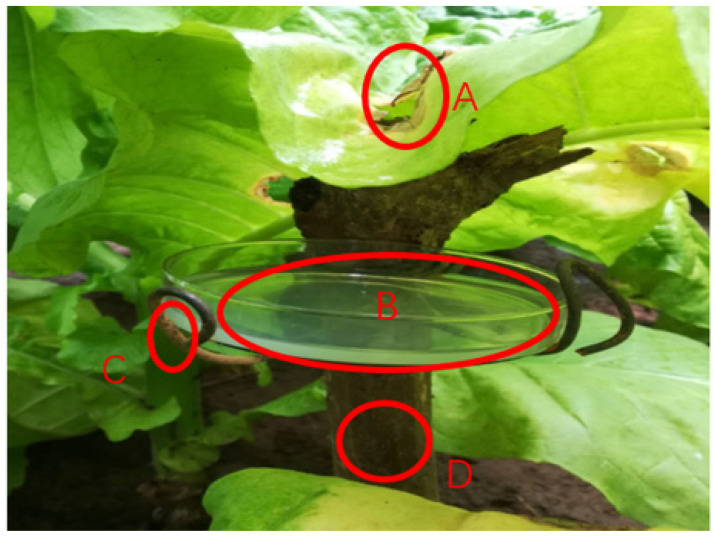
A simple device for collecting spores. (**A**) Diseased leaves in the field. (**B**) Water agar medium. (**C**) Fixed rigging. (**D**) Support tool for adjustment height.

A simple spore collection device (Figure 2) was used to collect the sexual spores induced by the aforementioned chemical fungicides. The device consisted of a culture dish containing agar medium placed 3–5 cm below a tobacco leaf with a white fruiting layer. To investigate the circadian rhythm of sexual sporulation, the spore stock solution diluted 10-fold and diluted 100-fold were inoculated onto young leaves of healthy tobacco during six time periods: 18:00–22:00 (4 h), 22:00–02:00 (4 h), 02:00–06:00 (4 h), 06:00–10:00 (4 h), 10:00–14:00 (4 h), and 14:00–18:00 (4 h). The experiment included six treatments with seven replicates each. Each time period was considered as one treatment, and three tobacco plants were inoculated in each treatment. Additionally, three leaves were inoculated on each tobacco plant, and sterilized water was used as a control. Disease leaves were collected at 6, 12, and 24 h after inoculation for each treatment, and the number of spores in a 1 cm^2^ area was recorded under a microscope.

In order to examine the correlation between the pinnacle of sexual sporulation and the prevailing conditions of temperature, humidity, and illumination, a sophisticated temperature and humidity recorder (Hobo, model U23) was positioned on the tobacco during the aforementioned experiment. Its purpose was to meticulously document the daily fluctuations in temperature and humidity over a continuous recording period of 15 days. By plotting the average variations in temperature and humidity across distinct time intervals, it became feasible to establish a direct correspondence between the recorded temperature and humidity levels and the spore count at each respective time frame. This analysis ultimately enabled the identification of the most conducive temperature and humidity conditions for optimal sexual sporulation.

### 2.3. The Infection Process of Sexual Spores

To elucidate the pathogenicity of sexual spores, the initial spore solution was carefully inoculated onto healthy central tobacco leaves. Subsequently, the solution was subjected to dilutions of 10-fold and 100-fold. As a control, sterilized water was used for inoculation. The experiment was meticulously designed with four distinct treatments and three replicates. Each treatment corresponded to a specific concentration of the inoculum. Within each treatment, three tobacco plants were inoculated, and three leaves were chosen for inoculation on each tobacco plant. Disease leaves were diligently collected at 2, 4, and 6 h post-inoculation for each treatment. Detailed observations were made regarding the symptoms of leaf disease, and the precise number of disease spots was recorded.

To clarify the spore survival time in vitro, the collected sexual spores were induced by propiconazole-morpholine guanidine during the night (18:00–06:00) and made into spore stock solution, using the same method as above. We inoculated the spore stock solution after standing for 1 h, 2 h, 4 h, and 6 h onto vigorous central tobacco leaves. The experiment was designed with 4 treatments and 6 replicates, each standing time of spore stock solution was treated as a treatment, 3 tobacco plants were inoculated in each treatment, and 3 leaves were inoculated on each tobacco plant. Collected disease leaves for each treatment, observed the symptoms of leaf disease and recorded the number of disease spots.

### 2.4. Biocontrol of Streptomyces rectiolaceus A8 against Tobacco Target Spot Disease

The experiments were conducted in fields previously affected by tobacco target spot disease. The trial followed a randomized design with four treatments and three replications. Each planting area measured 10 m × 1.4 m (50 planting holes) with a total of 12 districts. Two-month-old tobacco seedlings were transplanted into the field. To control the disease, *Streptomyces rectiolaceus* A8 was sprayed after the occurrence of natural tobacco leaf disease. The treatments were as follows: treatment A8-1 involved spraying the stock solution of A8 at a concentration of 265 mL/L; treatment A8-10 involved spraying the stock solution diluted by a factor of 10 (26.5 mL/L) of A8; treatment A8-100 involved spraying the stock solution diluted by a factor of 100 (2.65 mL/L) of A8; treatment CK served as the control, where an equal amount of water was sprayed. Each planting area was sprayed with 1 L of each concentration gradient of A8. After seven days, the biofungicide was reapplied at the same dosage to maintain its effectiveness. Disease incidence was investigated five days before and after the budding period, and six randomly selected diseased tobacco leaves were collected from each treatment group. Each tobacco leaf was stained with safranine and KOH dye; then, they were separately observed under a microscope to record the number of basidiospores, sporogenic structures, and fruiting bodies in a designated area measuring 1 cm × 1 cm. Disease incidence (Di), disease index (DI), disease incidence growth rate (D.G.r), disease index growth rate (D.G.R), and relative control effect (R.C.E.) were calculated using the following formulas:

Di (%) = (number of diseased leaves/total number of investigated leaves) × 100

DI = ∑(number of diseased leaves × disease grading scale)/(total number of investigated leaves × highest disease rating scale) × 100

D.G.r = (Di after the application A8 − Di before the application A8)/Di before the application A8

D.G.R = (DI after the application A8 − DI before the application A8)/DI before the application A8

R.C.E. (%) = (control D.G.R − treatment D.G.R)/control D.G.R × 100

### 2.5. Determination of Antibacterial Substances in Streptomyces rectiolaceus A8

Ethyl acetate was employed as the extraction agent and mixed with an equivalent volume of *Streptomyces rectiolaceus* A8 in a separate funnel. The mixture was vigorously shaken and allowed to stand for 15 min, permitting the collection of the upper organic phase in a conical flask. Then, 10 g of anhydrous sodium sulfate was added to facilitate overnight drying and dehydration. Vacuum rotational concentration was utilized for distillation and concentration under the following conditions: 45 °C, 80 rotations per minute, and a pressure of 0.6 MPa. The optimal distillation state was achieved when the recovery liquid delicately dripped down. The resulting concentrate was collected and quantified by supplementing it with 1000 μL of ethyl acetate. After undergoing two rounds of filtration via a needle filter, the sample was collected in a 1.5 mL brown sample bottle. The identification of antifungal compounds was performed using GC-MS.

### 2.6. Statistical Analyses

All statistical analyses were performed using Microsoft Excel 2021 and SPSS 26.0 software (IBM, Armonk, NY, USA), and GraphPad Prism 8.0 (GraphPad Prism Software Inc., San Diego, CA, USA) was used to plot the graphs. One-way ANOVA analysis was carried out to analyze the between-group variance (*p* < 0.05). Error bars indicated standard deviation (SD).

## 3. Results

### 3.1. Induction of Chemical Fungicides on the Sex Spores of R. solani AG-3 TB

**Figure 3:**
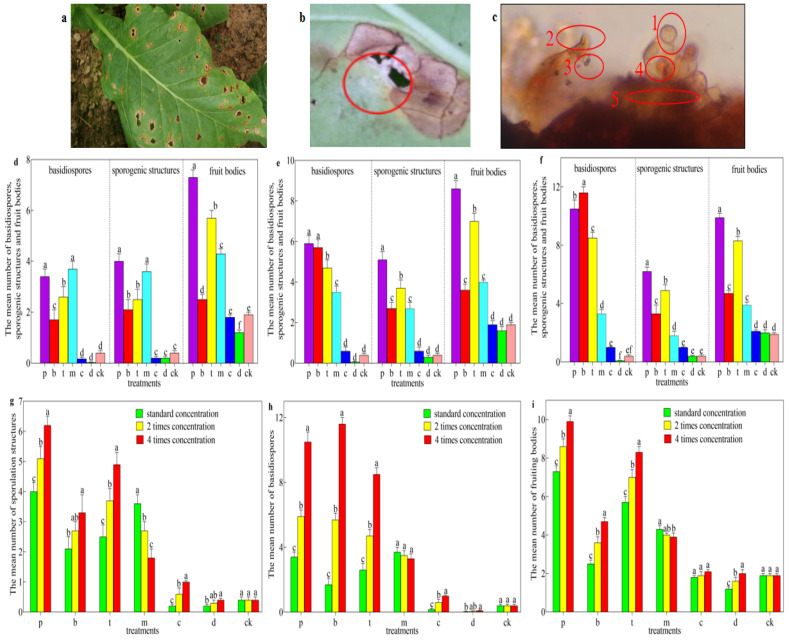
Induction effects of chemical fungicides on sexual spores. (**a**) The diseased leaves formed light brown lesions. (**b**) The diseased leaves formed a white fruiting layer, which has been circled in red. (**c**) Sexual spore structure observed under the microscope in tobacco target spot disease: 1 and 3, basidiospore; 2 and 4, sporulation structure; 5, fruiting bodies. (**d**) The number of basidiospores, sporulation structure and fruiting bodies treated with a variety of fungicides at regular concentrations. (**e**) The number of basidiospores, sporulation structure and fruiting bodies treated with various fungicides at 2 times the usual concentration. (**f**) The number of basidiospores, sporulation structure and fruiting bodies treated with various fungicides at 4 times the usual concentration. (**g**) Number of basidiospores treated with different concentrations of various fungicides. (**h**) Number of sporulation structures treated with different concentrations of various fungicides. (**i**) Number of fruiting bodies treated with various concentrations of various fungicides. Here, p: propiconazole-morpholine guanidine. b: bordeaux solution. t: thiophanate-methyl. m: mancozeb. c: carbendazim. d: dimethomorph. ck: control. Error bars represent SD. One-way ANOVA followed by the Tukey–Kramer test was performed (*p* < 0.05). Different letters indicate significant differences, while the same letters indicate non-significant differences in the figure caption. Same below all.

In the field, the disease manifested as the formation of light brown lesions (Figure 3a) or a white fruiting layer (Figure 3b) on tobacco leaves. Microscopic examination revealed the following spore structures associated with the disease: 1 and 3, basidiospores; 2 and 4, sporulation structure; 5, fruiting bodies (Figure 3c). Additionally, compared to the control group, the use of the four fungicides significantly increased the numbers of basidiospores, sporulation structures, and fruiting bodies. At the conventional concentration, the number of basidiospores treated with mancozeb was the highest, at 3.7, and the number of sporulation structures and fruiting bodies treated with propiconazole-morpholine guanidine were the highest, which were 4.0 and 7.3, respectively (Figure 3d). At two times the conventional concentration, the number of basidiospores, sporulation structures, and fruiting bodies after the treatment of propiconazole-morpholine guanidine was the highest, which were 5.9, 5.1, and 8.6, respectively (Figure 3e). At four times the conventional concentration, the number of basidiospores treated with bordeaux solution was the highest, at 11.6, and the number of sporulation structures and fruiting bodies treated with propiconazole-morpholine guanidine were the highest, which were 6.2, and 9.9, respectively (Figure 3f). However, there was no significant difference in the number of basidiospores, sporulation structures, and fruiting bodies between the carbendazim and dimethomorph treatments and the control group; the number of basidiospores, sporulation structures, and fruiting bodies of the control group were 0.4, 0.4, and 1.9, respectively (Figure 3d–f). It can be seen that the four fungicides have a significant effect on the induction of sexual sporulation of *R. solani* AG-3TB. Among them, the number of basidiospores, sporulation structures, and fruiting bodies were significantly different and increased in turn with the increase in the concentration of the first three fungicides, but the number of basidiospores, sporulation structures, and fruiting bodies decreased in turn with the increase in the concentration of mancozeb (Figure 3g–i).

### 3.2. Circadian Rhythm and Conditions of Sexual Sporulation

**Figure 4:**
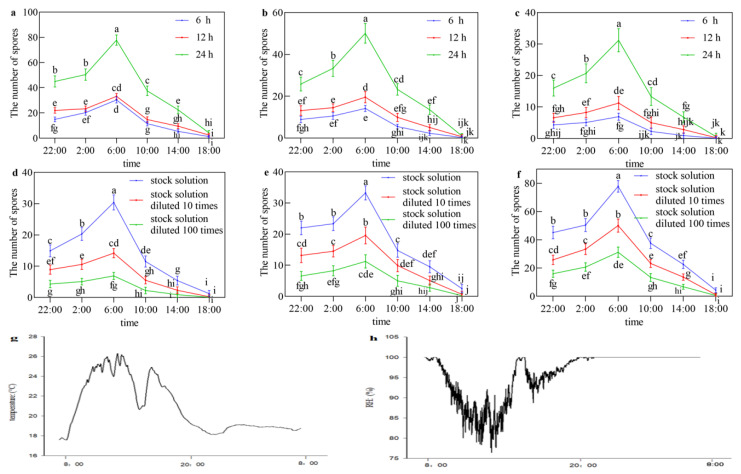
Circadian rhythm and conditions of sexual sporulation. (**a**) The number of spores produced at 6, 12, and 24 h after inoculation with a stock solution of sexual spores collected during different time periods. (**b**) The number of spores produced at 6, 12, and 24 h after inoculation with stock solution diluted 10-fold of sexual spores collected during different time periods. (**c**) The number of spores produced at 6, 12, and 24 h after inoculation with stock solution diluted 100-fold of sexual spores collected during different time periods. (**d**) The number of spores produced at 6 h after inoculation with stock solution, stock solution diluted 10-fold and stock solution diluted 100-fold of sexual spores collected during different time periods. (**e**) The number of spores produced at 12 h after inoculation with stock solution, stock solution diluted 10-fold and stock solution diluted 100-fold of sexual spores collected during different time periods. (**f**) The number of spores produced at 24 h after inoculation with stock solution, stock solution diluted 10-fold and stock solution diluted 100-fold of sexual spores collected during different time periods. (**g**) The temperature corresponding to the peak sporulation period. (**h**) The relative humidity corresponding to the peak sporulation period.

The number of spores produced at 6, 12, and 24 h after inoculation with different dilutions of a suspension sexual spores collected at different time periods exhibits significant variations. Specifically, the spore count at 24 h post-inoculation is significantly higher compared to that at 6 and 12 h post-inoculation. The increase in spore count suggests that longer inoculation times result in higher spore production. Moreover, as the duration of spore inoculation increases, the disparity in spore numbers between different time intervals also becomes more pronounced (Figure 4a–c). Moreover, there were significant differences in the number of spores produced after inoculation with stock solution, solution diluted 10-fold, and solution diluted 100-fold containing sexual spores collected at different time periods. These differences exhibited a decreasing trend, indicating that higher inoculation solution concentrations lead to greater spore yields. As the spore concentration is progressively diluted, the disparity in spore numbers between different concentration gradients also diminishes (Figure 4d–f). There were significant differences in the number of spores produced upon reinfection with sexual spores collected during six different time periods. Specifically, spore treatments collected and inoculated between 02:00 and 06:00 exhibited a significantly higher spore count compared to treatments collected and inoculated during the other five time periods. Spore treatments collected and inoculated between 14:00 and 18:00 yielded the lowest number of spores. The number of spores produced by treatments collected and inoculated between 18:00 and 06:00 gradually increased, while those collected and inoculated between 06:00 and 18:00 showed a gradual decrease. This indicates that spore production is more prominent during the night, displaying stronger pathogenicity, while spore production during the day is relatively minimal or non-existent (Figure 4a–f). The temperature, relative humidity, and lighting conditions corresponding to the peak sporulation period were approximately 19 ± 1 °C, nearly 100% humidity, and darkness, respectively (Figure 4g,h).

### 3.3. The Infection Process of Sex Spores of R. solani AG-3 TB

**Figure 5:**
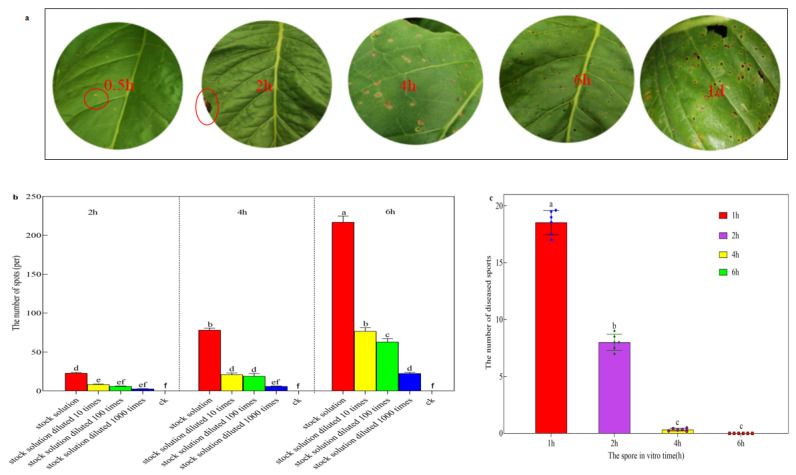
Infection law for sexual spores. (**a**) Symptoms in tobacco leaves at 0.5, 2, 4, and 6 h after inoculation with sexual spores, small circular lesion has been circled in red. (**b**) Number of lesions produced by tobacco leaves at 2, 4, and 6 h after inoculation with sexual spores. (**c**) The number of lesions produced on the tobacco leaf after inoculation with sexual spores cultured 1, 2, 4, and 6 h in vitro.

The disease progresses rapidly within 30 min of inoculation with the stock solution of sexual spores. Infected leaves exhibit pale yellow edges and small circular lesions in the center, measuring approximately 2–3 mm in diameter, with a white-yellow color. Within 2 h of inoculation, a significant number of white-yellow lesions become visible. Within 4 h of inoculation, these lesions expand to reach a diameter of 4–5 mm and change their color to yellowish-brown, which is accompanied by a few dark green lesions. After 6 h, numerous irregular dark green and yellowish-brown lesions appear, leading to the formation of perforations. Within a day, the dark green lesions disappear, and the typical lesions of tobacco target spot disease become evident. The yellowish-brown lesions on the inoculated leaves merge together, resulting in a large number of perforations (Figure 5a).

The number of diseased spots that formed on tobacco leaves at 2, 4, and 6 h after inoculation with different concentrations of sex spores varied significantly. In particular, the number of diseased spots formed on tobacco leaves at 6 h after inoculation was significantly higher than the number of diseased spots formed on tobacco leaves at 2 h and 4 h after inoculation. In addition, the number of diseased spots that formed on tobacco leaves after inoculation with the stock solution diluted 10-fold, diluted 100-fold, and diluted 1000-fold differed significantly. Specifically, the number of diseased spots that formed on tobacco leaves after inoculation with the stock solution was significantly higher than the number of diseased spots that formed on tobacco leaves after inoculation with the stock solution diluted 10-fold, 100-fold and 1000-fold. The number of diseased spots formed indicates that the longer the inoculation time, the more diseased spots are formed, and the difference in the number of lesions between the different time gradients increases as the spore inoculation time increases. The higher the concentration of inoculation solution, the more diseased spots formed, and with the continuous dilution of spore concentration, the difference in the number of lesions between different concentration gradients also decreased (Figure 5b).

After culturing sexual spores in vitro for 1, 2, 4, and 6 h and then inoculating them onto tobacco leaves, there was a significant difference in the number of lesions produced. As the culturing time of sexual spores in vitro increased, the number of formed lesions gradually decreased. Specifically, the highest number of lesions was observed after inoculating sexual spores cultured in vitro for 1 h, averaging 11.56 lesions. Conversely, the number of lesions on tobacco leaves reduced to the lowest after inoculating sexual spores cultured in vitro for 6 h, which was 0 lesions. This indicates that the effective survival time of sexual spores in vitro does not exceed 6 h (Figure 5c). 

### 3.4. Streptomyces rectiolaceus A8 Suppressed Tobacco Target Spot Disease

**Figure 6:**
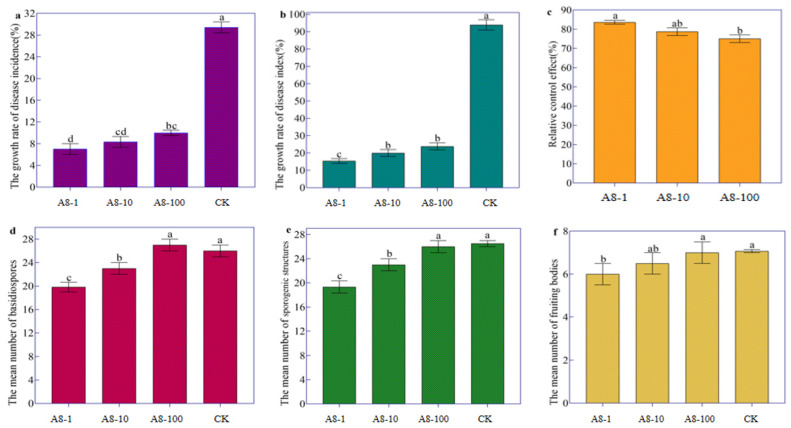
*Streptomyces rectiolaceus* A8 suppressed tobacco target spot disease caused by the sexual spores of *R. solani* AG-3TB. (**a**) The growth rate of disease incidence after treatment with various dilutions of A8. (**b**) The growth rate of disease index after treatment with various dilutions of A8. (**c**) Relative control effect after treatment with various dilutions of A8. (**d**) The number of basidiospores after treatment with various dilutions of A8. (**e**) The number of sporulation structure after treatment with various dilutions of A8. (**f**) The number of fruiting bodies after treatment with various dilutions of A8.

The growth rates of disease incidence and disease index after treatment with various dilutions of A8 fermented broth were significantly lower than in the control group. As A8 was continuously diluted, the growth rate of disease incidence and disease index increased, while the relative control effect decreased. Of these, the A8 stock solution had the lowest rate of disease incidence and disease index growth at 7.02 percent and 15.38 percent, respectively, while A8 stock solution had the highest relative control effect at 83.63 percent. However, the 10-fold and 100-fold dilution of the A8 showed control effects against tobacco target spot disease, which were lower than the treatment A8-1 (Table 1, Figure 6a–c).

Further study found that the number of basidiospores and sporulation structures of treatment A1 and A2 was significantly lower than that of the control, while the number of basidiospores and sporulation structures of treatment A3 was not significantly different from that of the control (Figure 6d,e); the number of fruiting bodies of treatment A1 was significantly lower than that of the control, and the number of fruiting bodies of treatment A2 and A3 was not significantly different from that of the control (Figure 6f). The results showed that the raw liquid of A8 fermenting broth significantly inhibited the production of sex spores and effectively controlled tobacco target spot disease. However, with the continuous dilution of the A8, the number of basidiospores, sporulation structures and fruiting bodies also increased, and the relative control effect became lower and lower.

Through GC-MS testing, A8 was found to contain 114 compounds, including 44 aromatic hydrocarbons, 18 esters, 13 fatty acids, 11 alkanes, 10 alcohols, 9 ketones, 6 phenols, 2 amides and 1 olefin. Four substances with antibacterial activity, phenylacetic acid, bis (2-ethylhexyl) phthalate, eicosane and 2, 4-di-tert-butylphenol, have been detected. In the literature, bis (2-ethylhexyl) phthalate and eicosane have strong antagonism against *R. solani* AG-3 (Supplementary Materials Table S1).

## 4. Discussion

The sexual spores are characterized by their light weight, large number, wide range of infection, rapid spread, and difficulty regarding prevention and control. It is the main means of tobacco target spot disease pathogen infection. *R. solani* AG-3TB does not produce any asexual spores, and the sexual spores (teliospores) are still hard to induce in vitro because the activity of sexual spores is very fragile [2]. There have been several reports of sexual spore induction in *Rhizoctonia*. For example, Chen used the soil induction method to induce the sexual generation of 30 *R. solani* strains distributed in five different anastomosis groups (AG-1, AG-2, AG-3, AG-4, AG-5), and only the test strains of two anastomosis groups (AG2-1, AG-4) produced sexual generation [31]. Jurick et al. used a medium-induced method to obtain the sexual spores of AG-1 [32,33]. Shew et al. conducted a study on plant tissue induction and discovered that after 5–6 days of inoculation at the tobacco stem base of the *R. solani* AG-2-2, a significant quantity of basidiospores was observed on the soil surface and plant stems. These basidiospores were occasionally found on both the front and back surfaces of the leaves [34]. These induction methods have induced the sexual spores of three different anastomosis groups (AG2-1, AG-4, AG2-2) of *Rhizoctonia*. In general, there are differences in the induction conditions of sexual spores in different anastomosing groups, and even within an anastomosing group, there are differences in the induction conditions of sexual spores in different strains. The sporulation ability of *R. solani* may be determined by the genotype of the fungus. Therefore, the results of sexual spore induction in such fungi are also unpredictable. It is only through continuous attempts and exploration that the most simple and effective method of sporulation suitable for the target strain can be found.

In general, six kinds of chemical fungicides, such as propiconazole-morpholine guanidine, bordeaux mixture, thiophanate-methyl, mancozeb, carbendazim, and dimethomorph, are widely used to control diseases. Among them, propiconazole is a systemic triazole fungicide that can prevent most fungal diseases [35]. Initially employed as the famous bordeaux mixture, this compound is commonly used in conventional agriculture to control oomycete diseases [36]. Thiophanate-methyl and carbendazim are commonly used to control peach diseases [37]. Mancozeb is commonly used to control tobacco diseases [38]. The application of dimethomorph is the main method to prevent the occurrence of plant diseases caused by *Phytophthora nicotianae* [39]. However, this study found that the application of four fungicides, such as propiconazole-morpholine guanidine, bordeaux mixture, thiophanate-methyl, and mancozeb, significantly promoted the production of the basidiospores, sporulation structure and fruiting bodies of *R. solani* AG-3TB, while the number of basidiospores, sporulation structure and fruiting bodies of *R. solani* AG-3TB treated with carbendazim and dimethomorph was not significantly different from that of the control. The results of the present study are not inconsistent with previous studies given the following points. First, studies have shown that the antagonistic ability of biocontrol bacteria against plant pathogens is not equivalent to the control ability [40]. Similarly, the rate of inhibition of disease-causing bacteria by chemical fungicides is not equivalent to control the capacity. Previous studies have been carried out on indoor plates to suppress the mycelium of the pathogen hyphae. However, this study was carried out in the field of tobacco target spot disease caused by the sexual spores of *R. solani* AG-3 TB. Second, sexual spores serve as a means of re-infection in the tobacco target spot disease cycle. In a nutritionally wealthy environment, fungi generally produce mitotic spores (the anamorphic state), but when conditions become unfavorable for vegetative growth, they may initiate sexual reproduction (teleomorphic state), and sexual spores of fungi can survive under adverse conditions [41,42,43]. As tobacco-targeted spot pathogens do not produce asexual spores, sexual spores are used as a means of re-infection in the disease cycle. This method of infection is relatively ad hoc and is particularly rare in *Rhizoctonia* disease. In a nutritionally rich environment, fungi generally produce mitotic spores (the anamorphic state), but when conditions become unfavorable for vegetative growth, they may initiate sexual reproduction (teleomorphic state). Therefore, when chemical fungicides act on the mycelia of tobacco target spot disease pathogens, they will inhibit the growth of mycelia, which may cause the pathogens to transform from asexual hyphae to sexual spores, while tobacco target spot disease pathogens can still produce sexual spores to infect tobacco plants in the field. The above two points indicate that the rate of inhibition of tobacco target spot disease by chemical fungicides is excellent, but the field control effect is not favorable. For example, the inhibition rate of 70% thiophanate-methyl to tobacco target spot pathogen was 88%, but the relative control effect in the field was only 42%, and the effect was not ideal [44].

Currently, domestic and international studies of the pathogenic mechanism of AG-3 have focused on the mycelial infection mechanism, while the mechanism of sporulation induced by fungicides is not well understood. In this study, the three fungicides of propiconazole-morpholine guanidine, thiophanate-methyl and bordeaux mixture with a favorable induction effect all contain the mineral element sulfur. It has been conjectured that the sulfur content is one of the factors responsible for the effective induction of spores by the fungicide. It is known that fungal cells and spores can rapidly take up elemental sulfur [45]. Li’s study showed that a higher concentration of SO_4_^2−^ had a significant effect on the induction of sporangia of Phytophthora nicotianae [46]. Excessive sulfur spraying on the leaves may enhance the plant’s photosynthetic capacity and increase photosynthetic products. Xie et al. showed that sulfur application could increase the leaf area and specific leaf weight of maize, increase the content of soluble protein and photosynthetic pigment in functional leaves, and increase the photosynthetic rate of leaves [47]. In this study, propiconazole-morpholine guanidine, bordeaux mixture, thiophanate-methyl, and mancozeb significantly promoted the production of sexual spores of *R. solani* AG-3TB. In particular, the number of spores decreases with increasing concentration of the mancozeb, and it increases with increasing concentration of the remaining three fungicides. The results of mancozeb treatment were opposite to those of propiconazole-morpholine guanidine, thiophanate-methyl, and bordeaux solution treatment. It is conjectured that Mn^2+^ and Zn^2+^ are responsible for this difference in behavior. Li also reported that a certain concentration range of Mn^2+^ could promote the induction of sporangia of *P. nicotianae*, and with the increase in its concentration, the induction effect of sporangia continued to weaken, and only a lower concentration of Zn^2+^ could promote the induction of sporangia of *P. nicotianae*, indicating that the effect of Zn^2+^ was special, and the effect of Mn^2+^ on sporulation was dominant [46].

Combining previous studies and this study, the results confirm that propiconazole -morpholine guanidine, bordeaux mixture, thiophanate-methyl and mancozeb are not applied to the field control of tobacco target spot disease, but they are applicable to the induction of sexual spores of *R. solani* AG-3 TB. Carbendazim and dimethomorph cannot be used to induce sexual spore production in AG-3, but whether they can be applied to the field control of AG-3 is subject to additional validation. At the same time, the results of this study perfectly explain the phenomenon in which tobacco farmers often use elevated concentrations and extreme doses of fungicides to cause outbreaks of disease in tobacco fields. It is worth noting that propiconazole, carbendazim, thiophanate and mancozeb are now banned in EU countries [47,48], but they are the most widely used fungicides and insecticides in China, and they are still the main products for farmers to protect crops [49,50]. It is well known that propiconazole-morpholine guanidine is a wettable powder with the following active ingredients and their concentrations: morpholine guanidine 16%, and propiconazole 2%. Propiconazole is a low-toxicity, systemic triazole fungicide that can effectively inhibit the synthesis of adenosine triphosphate (ATP) in pathogens, offering protective and therapeutic effects and inhibiting the germination of pathogen spores. Morpholine guanidine has both systemic and therapeutic actions; it is capable of entering the plant through water stomata, inhibiting or destroying the formation of nucleic acids and lipoproteins, preventing the replication process of viruses, and serving an antiviral function. When mixed together, they can be used to control tobacco virus diseases. Mancozeb (Man), a key contact protective fungicide belonging to the dithiocarbamates class, primarily works by inhibiting the oxidation of pyruvic acid inside fungal cells [51]. It decomposes easily under high temperature and humidity, exhibits low toxicity to higher animals but can cause irritation to human skin and mucous membranes. It should not be mixed with alkaline substances or copper formulations but can be combined with various insecticides, other fungicides, and acaricides. The use of this agent should be avoided at noon during the hot season. Methiobuzin is considered a high-efficiency, low-toxicity, low-residue systemic fungicide with both protective and therapeutic effects [52]. It can be mixed with alkaline agricultural chemicals such as lime sulfur mixture but should not be mixed with copper formulations and is not recommended for prolonged solo use. The acceptable daily intake (ADI) percentage of thiophanate-methyl ranges between 21.1% and 84.9%, and its acute reference dose (ARfD) percentage ranges between 2.1% and 19.2%, indicating a low risk to the Chinese population from the use of thiophanate-methyl [53].

In this study, the observation of sporulation rhythm and the records of temperature and humidity found that *R. solani* AG-3 TB produced more sexual spores during the night than during the day, and the number of sexual sporulation was most at 6:00 a.m. and least at 18:00 p.m., and the temperature, relative humidity, and illumination condition corresponding to the peak sporulation period were 19 ± 1 °C, approximately 100%, and dark, respectively. Light plays an essential role in inducing asexual sporulation (conidia) whereas in the absence of light, fungus initiates the formation of sexual fruiting bodies (cleistothecia) containing sexual spores (ascospores) [54]. Windels and Naito et al. successfully induced the basidiospores of AG-2-3 by separating the hyphae of soybean leaf blight and adding water in the box to maintain a high relative humidity and temperature of about 25 °C [55,56]. Adams, Whitney, and others reported that the sexual spores of *R. solani* are produced spores in the dark. When the relative humidity (RH) reached 97% and 99%, AG-1 and AG-4 produced abundant basidiospores, but under the condition of RH = 85.7%, no basidiospores or fruiting bodies were produced, and there were humidity differences and strong humidity conditions [57]. The sporulation rhythm and conditions of *R. solani* AG-3 TB in this study are similar to those of different fungi. This study complements and enriches previous studies on the conditions under which fungi produce sex spores. Conditions for vegetative hyphal growth and spore reproduction in fungi are different. In general, the conditions for spore production are more stringent than those for mycelial growth, and those for sexual spores are more stringent than for asexual spores.

In this study, spore suspension inoculation experiments were performed at different concentrations to determine the ability of the spore to infect and the time of in vitro survival using the number of lesions produced by the leaves as an indicator. The results showed that tobacco leaves were rapidly infected within half an hour of being inoculated with the spore stock solution. The infection of sexual spores was different from that of hyphae, the infection of hyphae was relatively slow, and the lesions appeared after 2 days [58]. In the presence of high concentrations of sexual spores or long inoculation times, faster and more numerous lesions are produced. However, the sexual spores fell off at the site of the lesion and were inoculated into the leaves of healthy tobacco plants after 6 h of in vitro, with no lesion present in the leaves, indicating that the maximum survival time of the sexual spores was not more than 6 h. The characteristics of rapid infection and severe incidence of sexual spores have become difficulties in the prevention and treatment of the disease [59]. At present, there are few reports on the infectivity, in vitro survival time, and host range of AG-3 sex spores.

The purpose of inducing sexual spore production in *R. solani* AG-3 TB by chemical fungicides and clarifying the laws of sexual spore prevalence is to better control the tobacco-targeted spot disease caused by sexual spores in *R. solani* AG-3 TB. The containment of *R. solani* in tobacco is difficult. Using microorganisms to control plant disease is a sustainable alternative to conventional fungicides [60]. The ability of *Streptomyces* species to produce plant-protective substances, such as enzymes, secondary metabolites, and volatile organic compounds, as well as their ability to induce plant immunity to respond to pathogens rapidly, indicate that they are excellent candidates as biocontrol agents [61]. In this study, *Streptomyces recall* A8 was found to be a potential biocontrol agent against tobacco target spot disease, with A8 being able to significantly inhibit the production of sex spores of *R. solani* AG-3 TB, with a control effect reaching 83.63% in field experiments. In addition, using GC-MS detection discovered that four substances with antibacterial activity, phenylacetic acid, bis (2-ethylhexyl) phthalate, eicosane and 2, 4-di-tert-butylphenol have been detected in A8. Studies have shown that bis (2-ethylhexyl) phthalate and eicosane have strong antagonism against *R. solani* AG-3 [62]. Similar findings have been reported in other crops, which also demonstrate similar findings. For example, the application of actinomycete XF significantly reduced the disease indices of stripe rust by 53.83% in the field [63]. *Streptomyces pratensis* S10 was reported to significantly inhibit the growth of Fusarium graminearum and effectively control wheat scab and decrease deoxynivalenol content [27]. Combining previous studies and this study, the results confirm that *Streptomyces rectiolaceus* strain A8 is a potential biocontrol agent for managing tobacco target spot disease.

## 5. Conclusions

This study found that four fungicides unexpectedly induced sexual spore production in *R. solani* AG-3 TB, which is the fungus causing tobacco target spot. Optimal conditions for spore formation were night time, 19 ± 1 °C, 100% humidity, and darkness, with spores surviving less than 6 h in vitro. One compound, A8, inhibited spore formation and showed an 83.63% control efficacy, containing bis(2-ethylhexyl) phthalate and eicosane as active substances. These insights suggest that further research on fungicide use in disease management is required. 

## Figures and Tables

**Table 1:** The effect of A8 on diseases.

Treatments	Disease Incidence (%)		Disease Index (%)	
	Before the Application of A8	After the Application of A8	Before the Application of A8	After the Application of A8
A8-1	57.00	61.00	2.60	3.00
A8-10	60.00	65.00	3.50	4.20
A8-100	70.00	77.00	4.20	5.20
CK	52.00	67.30	3.30	6.40

## Data Availability

Data are contained within the article and Supplementary Materials.

## Supplementary Materials

The following supporting information can be downloaded at https://www.mdpi.com/article/10.3390/life14030404/s1, Table S1. GC-MS analysis results of A8.
