# Supplementary material for: Effects of Chemical and Biological Fungicide Applications on Sexual Sporulation of Rhizoctonia solani AG-3 TB on Tobacco
Source: Life (Basel). 2024 Mar 18;14(3):404. doi: 10.3390/life14030404 (PMC10971793; doi:10.3390/life14030404)
Supplement: Supplementary file 1 [file life-14-00404-s001.zip › life-2899936-supplementary.pdf]

Table S1. GC-MS analysis results of A8 FL.

Name	Molecular formula	Similarity	Molecular weight	Classification
naphthalene	$C_{10}H_8$	98	128	aromatic hydrocarbons
paraxylene	C_8H_{10}	98	106	aromatic hydrocarbons
1,2,3,5-tetramethylbenzene	$C_{10}H_{14}$	98	134	aromatic hydrocarbons
palmitoleic acid	$C_{16}H_{30}O_2$	97	254	fatty acids
2-dimethylbenzene	C_8H_{10}	97	106	aromatic hydrocarbons
dibutylphthalate	$C_{16}H_{22}O_4$	97	278	ester
2-Ethyl-1,4-dimethylbenzene	$C_{10}H_{14}$	97	134	aromatic hydrocarbons
1-Ethyl-3-methylbenzene	C_9H_{12}	97	120	aromatic hydrocarbons
1-Ethyl-2,4-dimethylbenzene	$C_{10}H_{14}$	97	134	aromatic hydrocarbons
1-Methyl-3-(1-methylethyl) -benzene	$C_{10}H_{14}$	97	134	aromatic hydrocarbons
1,2,4,5-tetramethylbenzene	$C_{10}H_{14}$	97	134	aromatic hydrocarbons
1,2,3,5-tetramethylbenzene	$C_{10}H_{14}$	97	134	aromatic hydrocarbons
1,2,3,4-tetramethylbenzene	$C_{10}H_{14}$	97	134	aromatic hydrocarbons
n-hexadecanoic acid	$C_{16}H_{32}O_2$	96	256	fatty acids
mesitylene	C_9H_{12}	96	120	aromatic hydrocarbons
3 furan methanol	$C_5H_6O_2$	96	98	alcohols
2-Ethyl-1,4-dimethylbenzene	$C_{10}H_{14}$	96	134	aromatic hydrocarbons
2-methylnaphthalene	$C_{11}H_{10}$	96	142	aromatic hydrocarbons
1-Ethyl-3,5-dimethylbenzene	$C_{10}H_{14}$	96	134	aromatic hydrocarbons
1-Ethyl-2-methylbenzene	C_9H_{12}	96	120	aromatic hydrocarbons
1-Methyl-3- (1-methylethyl) -benzene	$C_{10}H_{14}$	96	134	aromatic hydrocarbons
1,2,3-Trimethylbenzene	C_9H_{12}	96	120	aromatic hydrocarbons
1,2,3,5-Tetramethylbenzene	$C_{10}H_{14}$	96	134	aromatic hydrocarbons
Indene	C_9H_{10}	95	118	aromatic hydrocarbons
pentadecanoic acid	$C_{15}H_{30}O_2$	95	242	fatty acids
diisocapryl phthalate	$C_{24}H_{38}O_4$	95	390	ester
paraxylene	C_8H_{10}	95	106	aromatic hydrocarbons
propylbenzene	C_9H_{12}	95	120	aromatic hydrocarbons
2,3-Dihydro-5-methyl-1H-indene	$C_{10}H_{12}$	95	132	aromatic hydrocarbons
2- (Dodecyloxy) -ethanol	$C_{14}H_{30}O_2$	95	230	alcohols
2- (2-methylphenyl) ethylene oxide	$C_9H_{10}O$	95	134	alkanes
1-Methyl-4- (1-methylpropyl-benzene)	$C_{11}H_{16}$	95	148	aromatic hydrocarbons
1-Methyl-3-propylbenzene	$C_{10}H_{14}$	95	134	aromatic hydrocarbons
1,3-Dimethylbenzene	C_8H_{10}	95	106	aromatic hydrocarbons
(z) Oleamide	$C_{18}H_{35}NO$	95	281	amides
(1-methylpropyl) -benzene	$C_{10}H_{14}$	95	134	aromatic hydrocarbons
N-octadecyl acetate	$C_{20}H_{40}O_2$	94	312	ester
ethylene glycol diacetate	$C_{22}H_{44}O_2$	94	340	ester
myristic acid	$C_{14}H_{28}O_2$	94	228	fatty acids
Nonadecyl heptafluorobutyrate	$C_{23}H_{39}F_7O_2$	94	480	ester

o-cymene	$C_{10}H_{14}$	94	134	aromatic hydrocarbons
dimethylphthalate	$C_{10}H_{10}O_4$	94	194	ester
1-heptacosanol	$C_{27}H_{56}O$	94	396	alcohols
3-hydroxydecanoic acid (3hd)	$C_{10}H_{20}O_3$	94	188	fatty acids
2-Ethyl-1,3-dimethylbenzene	$C_{10}H_{14}$	94	134	aromatic hydrocarbons
2,3-Dihydro-4-methyl-1 (H) -indene	$C_{10}H_{12}$	94	132	aromatic hydrocarbons
1-Vinyl-4-ethylbenzene	$C_{10}H_{12}$	94	132	aromatic hydrocarbons
1-Ethyl-2,4-dimethylbenzene	$C_{10}H_{14}$	94	134	aromatic hydrocarbons
1-Methyl-4-propylbenzene	$C_{10}H_{14}$	94	134	aromatic hydrocarbons
1-Methyl-2-propylbenzene	$C_{10}H_{14}$	94	134	aromatic hydrocarbons
1,2-Benzenedicarboxylic acid, bis (2-methylpropyl) ester	$C_{16}H_{22}O_4$	94	278	ester
phthalate (2-ethylhexyl) phthalate	$C_{24}H_{38}O_4$	93	390	ester
Phthalic acid, hexyl-3-isobutyl ester	$C_{18}H_{26}O_4$	93	306	ester
Phthalic acid, hept-3-yl isobutyl ester	$C_{19}H_{28}O_4$	93	320	ester
Nonadecyl acetate	$C_{21}H_{42}O_2$	93	326	ester
2 ', 4 ' -Dihydroxy-3 ' -methylacetophenone	$C_9H_{10}O_3$	93	166	ketone
1-Methyl-4-propylbenzene	$C_{10}H_{14}$	93	134	aromatic hydrocarbons
(9E) -9-hexadecacarbon	$C_{26}H_{52}$	93	364	alkanes
pentamethylbenzene	$C_{11}H_{16}$	92	148	aromatic hydrocarbons
Cis-10-heptadecaenoic acid	$C_{17}H_{32}O_2$	92	268	fatty acids
Phthalic acid, bis (7-methyloctyl) ester	$C_{26}H_{42}O_4$	92	418	ester
cyclopropylbenzene	C_9H_{10}	92	118	aromatic hydrocarbons
2-acetyl-resorcinol	$C_8H_8O_3$	92	152	phenols
2,2 ' -Methylenebis (6-tert-butyl-4-methylphenol)	$C_{23}H_{32}O_2$	92	340	phenols
1-Methyl-2-propylbenzene	$C_{10}H_{14}$	92	134	aromatic hydrocarbons
1,4-Diethyl-2-methylbenzene	$C_{11}H_{16}$	92	148	aromatic hydrocarbons
1,3-diethylbenzene	$C_{10}H_{14}$	92	134	aromatic hydrocarbons
(1-methylpropyl) -benzene	$C_{10}H_{14}$	92	134	aromatic hydrocarbons
Tetrapentadecane	$C_{54}H_{110}$	91	758	alkanes
Phthalic acid, bis (7-methyloctyl) ester	$C_{26}H_{42}O_4$	91	418	ester
Adipic acid, bis (2-ethylhexyl) ester	$C_{22}H_{42}O_4$	91	370	ester
Diisooctyl adipate	$C_{22}H_{42}O_4$	91	370	ester
N- (2-phenylethyl) -acetamide	$C_{10}H_{13}NO$	91	163	amides
7-Ethyl-1,3,5-cycloheptatriene	C_9H_{12}	91	120	alkenes
2-Ethyl-1,4-dimethylbenzene	$C_{10}H_{14}$	91	134	aromatic hydrocarbons
2-Hydroxy-cyclopentadecanone	$C_{15}H_{28}O_2$	91	240	ketone
2-furanemethanol	$C_5H_6O_2$	91	98	alcohols
2- (hexadecyloxy) -ethanol	$C_{18}H_{38}O_2$	91	286	alcohols
1,2-Benzenedicarboxylic acid, dinonyl ester	$C_{26}H_{42}O_4$	91	418	ester
Cis-acetic acid	$C_{18}H_{34}O_2$	90	282	fatty acids

5-methyl-resorcinol	$C_7H_8O_2$	90	124	phenols
3-methyl-1,2-cyclopentanedione	$C_6H_8O_2$	90	112	ketone
3,4-Dimethylisopropylbenzene	$C_{11}H_{16}$	90	148	aromatic hydrocarbons
2-bromohexane	$C_6H_{13}Br$	90	164	alkanes
2-Hydroxy-cyclopentadecanone	$C_{15}H_{28}O_2$	90	240	ketone
2- (Tetradecyloxy) -ethanol	$C_{16}H_{34}O_2$	90	258	alcohols
1- (2,5-dihydroxyphenyl) -ethanone	$C_8H_8O_3$	90	152	ketone
n-hexadecanoic acid	$C_{16}H_{32}O_2$	89	256	fatty acids
n-tetratriacontane	$C_{34}H_{70}$	89	478	alkanes
n-hexatriacontane	$C_{36}H_{74}$	89	506	alkanes
pentamethylbenzene	$C_{11}H_{16}$	89	148	aromatic hydrocarbons
hendecanoic acid	$C_{11}H_{22}O_2$	89	186	fatty acids
L- (+) -ascorbic acid 2,6-hexacosate	$C_{38}H_{68}O_8$	89	652	ester
3,4-Dimethylisopropylbenzene	$C_{11}H_{16}$	89	148	aromatic hydrocarbons
2,4-Diethyl-1-methylbenzene	$C_{11}H_{16}$	89	148	aromatic hydrocarbons
1-Ethyl-4- (1-methylethyl) -benzene	$C_{11}H_{16}$	89	148	aromatic hydrocarbons
1-Ethyl-3,5-dimethylbenzene	$C_{10}H_{14}$	89	134	aromatic hydrocarbons
1-Ethyl-2,3-dimethyl-benzene	$C_{10}H_{14}$	89	134	aromatic hydrocarbons
1-Methyl-4- (1-methylpropyl) benzene	$C_{11}H_{16}$	89	148	aromatic hydrocarbons
1-Methyl-4- (1-methylpropyl) benzene	$C_{11}H_{16}$	89	148	aromatic hydrocarbons
1,3-Dimethyl-5- (1-methylethyl) -benzene	$C_{11}H_{16}$	89	148	aromatic hydrocarbons
(R) -6- (phenylmethyl) -4,7-diazaspiro [2.5] octane-5,8-dione	$C_{13}H_{14}N_2O_2$	89	230	ketone
(11Z) -11-pentadecene-1-ol	$C_{15}H_{30}O$	89	226	alcohols
Tetrahydro-2 (H) -pyran-2-methanol	$C_6H_{12}O_2$	88	116	alcohols
hexatriacontane	$C_{36}H_{74}$	88	506	alkanes
Phthalic acid, bis (7-methyloctyl) ester	$C_{26}H_{42}O_4$	88	418	ester
2-Hydroxy-3-methyl-2-cyclopenten-1-one	$C_6H_8O_2$	88	112	ketone
2-methyl-1,3-benzenediol	$C_7H_8O_2$	88	124	phenols
1-Ethyl-3- (1-methylethyl) -benzene	$C_{11}H_{16}$	88	148	aromatic hydrocarbons
1,2-Benzenedicarboxylic acid, dinonyl ester	$C_{26}H_{42}O_4$	88	418	ester
tetratetracontane	$C_{44}H_{90}$	87	618	alkanes
dodecanoic acid	$C_{12}H_{24}O_2$	87	200	fatty acids
eicosane	$C_{20}H_{42}$	87	282	alkanes
Dichloroacetic acid, trideca-2-alkynyl ester	$C_{15}H_{24}Cl_2O_2$	87	306	ester
3,4-Dimethylisopropylbenzene	$C_{11}H_{16}$	87	148	aromatic hydrocarbons
3,4-Dimethylisopropylbenzene	$C_{11}H_{16}$	87	148	aromatic hydrocarbons
2-Hydroxydecanoic acid	$C_{10}H_{20}O_3$	87	188	fatty acids
2-Methyl-1,4-benzenedialdehyde	$C_9H_8O_2$	87	148	aromatic aldehydes

2,4-di-tert-butylphenol	$C_{14}H_{22}O$	87	206	phenols
1-Methyl-4- (1-methylpropyl) benzene	$C_{11}H_{16}$	87	148	aromatic hydrocarbons
1,3-Diethyl-5-methylbenzene	$C_{11}H_{16}$	87	148	aromatic hydrocarbons
1,2-Benzenedicarboxylic acid, dinonyl ester	$C_{26}H_{42}O_4$	87	418	ester
1- (2-hydroxy-4-methoxyphenyl) -ethanone	$C_9H_{10}O_3$	87	166	ketone
Cis-9-hexadecene	$C_{16}H_{30}O$	86	238	fatty acids
dodecanoic acid	$C_{12}H_{24}O_2$	86	200	fatty acids
tetratriacontane	$C_{34}H_{70}$	86	478	alkanes
Phthalic acid, bis (7-methyloctyl) ester	$C_{26}H_{42}O_4$	86	418	ester
dicumyl peroxide	$C_{18}H_{22}O_2$	86	270	aromatic hydrocarbons
7-Hexadecen-1-ol	$C_{16}H_{30}O$	86	238	alcohols
7- (2-octyl-1-cyclopropene-1-yl) -1-heptanol	$C_{18}H_{34}O$	86	266	alcohols
4-Methyl-1,2-benzenediol	$C_7H_8O_2$	86	124	phenols
3-hydroxy myristic acid	$C_{14}H_{28}O_3$	86	244	fatty acids
3-Methyl-2,5-furandione	$C_5H_4O_3$	86	112	ketone
2 ' -Hydroxy-4 ' -methoxyacetophenone, acetate	$C_{11}H_{12}O_4$	86	208	ester
2 ', 4 ' -dimethoxyacetophenone	$C_{10}H_{12}O_3$	86	180	ketone
2,3-Dihydro-4,7-dimethyl-1H-indene	$C_{11}H_{14}$	86	146	aromatic hydrocarbons
2,2,4,6,6-pentamethyl-heptane	$C_{12}H_{26}$	86	170	alkanes
1-Ethyl-4- (1-methylethyl) -benzene	$C_{11}H_{16}$	86	148	aromatic hydrocarbons
1-Ethyl-2,4,5-trimethylbenzene	$C_{11}H_{16}$	86	148	aromatic hydrocarbons
1,3-Dimethyl-5- (1-methylethyl) -benzene	$C_{11}H_{16}$	86	148	aromatic hydrocarbons
(1,1-dimethylpropyl) -benzene	$C_{11}H_{16}$	86	148	aromatic hydrocarbons
